# Rage induces hepatocellular carcinoma proliferation and sorafenib resistance by modulating autophagy

**DOI:** 10.1038/s41419-018-0329-z

**Published:** 2018-02-14

**Authors:** Jun Li, Peng-Wen Wu, Yuan Zhou, Bo Dai, Peng-Fei Zhang, Yu-Hen Zhang, Yang Liu, Xiao-Lei Shi

**Affiliations:** Department of Hepatobiliary Surgery, Affiliated Drum Tower Hospital of Nanjing University Medical School, 321, Zhongshan Road, 210008 Nanjing, Jiangsu Province China

## Abstract

The receptor for advanced glycation end products (Rage) is involved in the development of various tumors and acts as an oncogenic protein. Rage is overexpressed in tumors including hepatocellular carcinoma (HCC). However, the molecular mechanism of Rage in HCC progression and sorafenib resistance remains unclear. In this study, enhanced Rage expression is highly associated proliferation and contributes to sorafenib resistance. Rage deficiency contributed to autophagy induction through activating AMPK/mTOR signaling pathway, which is important for sorafenib response. Moreover, the interactions between Rage and Rage ligands such as high mobility group box 1 (HMGB1) and s100a4 positively increased Rage expression. Our data indicate that Rage may be a potential target for therapeutic intervention in HCC and biomarker for sorafenib resistance.

## Introduction

Hepatocellular carcinoma (HCC) is the fifth common malignancy and the third main cause of cancer-associated death worldwide^1^. Since the high rate of vascular invasion, metastasis, and recurrence following curative liver resection, the prognosis of patients with HCC is extremely poor and the 5-year survival rate is no more than 40%^2^. Sorafenib has been used as the first-line systematic therapy for advanced HCC patients and improves 3 months survival^3^. However, the response rate of sorafenib treatment is very low and the molecular mechanism involved in sorafenib resistance has not been well elucidated. Therefore, it is imperative to find novel targets in HCC development and improve HCC response to sorafenib therapy.

Autophagy is a highly conserved catabolic process for removing and recycling damaged intracellular organelles^4^. Autophagy has been reported to play a critical role in many diseases including tumors^5–7^. Stress-induced autophagy such as nutrient deprivation could promote tumor cell survival, but excessive autophagy also lead to cell injury and apoptosis^8^. Accumulating data indicate autophagy is the leading cause for sorafenib resistance. Recently, Wu et al. reveal that the beta-2 adrenergic receptor contributes to autophagy inhibition through disrupting Beclin1/VPS34/Atg14 complex in an Akt-dependent manner, which accounts for sorafenib chemoresistance^9^. AMPK/mTOR signaling pathway has been demonstrated to be important in autophagy activation. Additionally, dysregulation of AMPK/mTOR is involved in sorafenib resistance^10^.

The receptor for advanced glycation end products (Rage) is usually expressed in diverse types of cells^11^. Rage has been demonstrated in the progression of many inflammatory diseases including tumors^11–13^. The interactions between Rage and its ligands, such as high mobility group box 1 (HMGB1) and s100 protein family, stimulate the activation of NF-kappaB, MAPK, and Akt/mTOR signaling pathways^12,14^. Recent studies indicate the relationship between Rage and autophagy^15,16^. However, the role of Rage in HCC development and sorafenib resistance has been rarely reported.

In our present study, Rage was highly expressed in HCC and promoted the proliferation of HCC cells. Reduced Rage expression improved sorafenib response by increasing autophagy in an AMPK/mTOR-dependent way. Moreover, interactions of Rage and Rage ligands were responsible for Rage expression, which contributed to the repression of sorafenib response. Together, we suggest Rage could be a novel target for HCC treatment and improvement of sorafenib resistance.

## Results

### Rage is overexpressed in HCC tissues

**Fig. 1:**
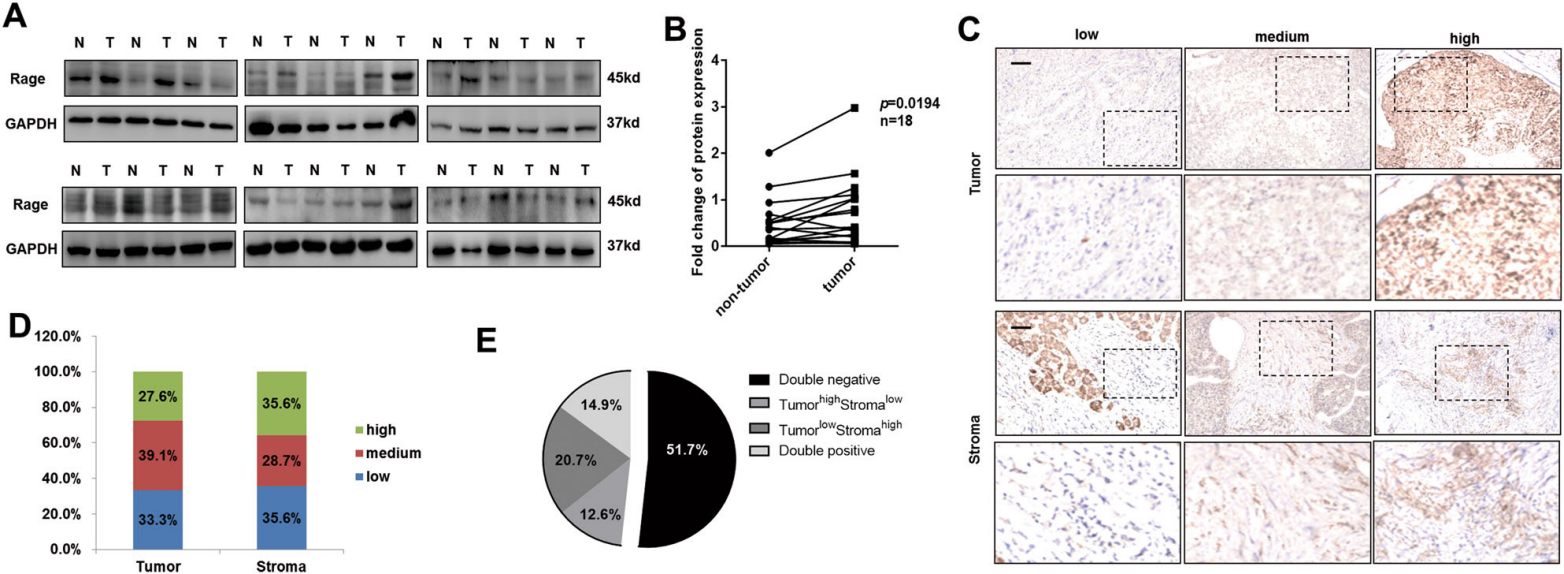
Rage expression is elevated in HCC. **a** Immunoblot analysis shows that Rage expression is higher in tumors than non-tumors. **b** Protein levels of Rage were normalized to GAPDH and analyzed. **c** IHC of Rage in HCC samples. Representative images show different intensities of staining of Rage in tumor cells and stromal cells. Scale bars, 100 μm. **d**,** e** Different intensities of staining of Rage in tumor cells and stromal cells were analyzed. Data are mean ± SEM, *p < 0.05, **p < 0.01, ***p < 0.001 by unpaired Student’s *t*-test

To determine the role of Rage in HCC progression, we initially evaluate Rage expression in 18 HCC specimens. We found that Rage protein expression was significantly higher in tumors compared to non-tumors (Fig. 1a, b). Moreover, we examined the expression of Rage in 68 paraffin-fixed HCC tissues (Supplementary Table S1). We observed different levels of Rage were expressed both in HCC cells and stromal cells (Fig. 1c–e). 14.9% of patients were Rage^high^ in both tumor cells and stromal cells. 51.7% of patients were Rage^low^ in both tumor cells and stromal cells. To demonstrate the clinical relevance of Rage, Rage expression of 68 HCC tissues was quantified. Compared to non-tumor tissues, Rage expression in tumors was the highest, which further confirmed the results of protein levels (Fig. S1A–B). For further analysis, patients were seprated into two groups with high and low Rage expression groups. We found that Rage expression was statistically associated with TNM stage, which suggested that Rage played an important role in HCC progression.

### High expression of Rage promotes HCC proliferation

**Fig. 2:**
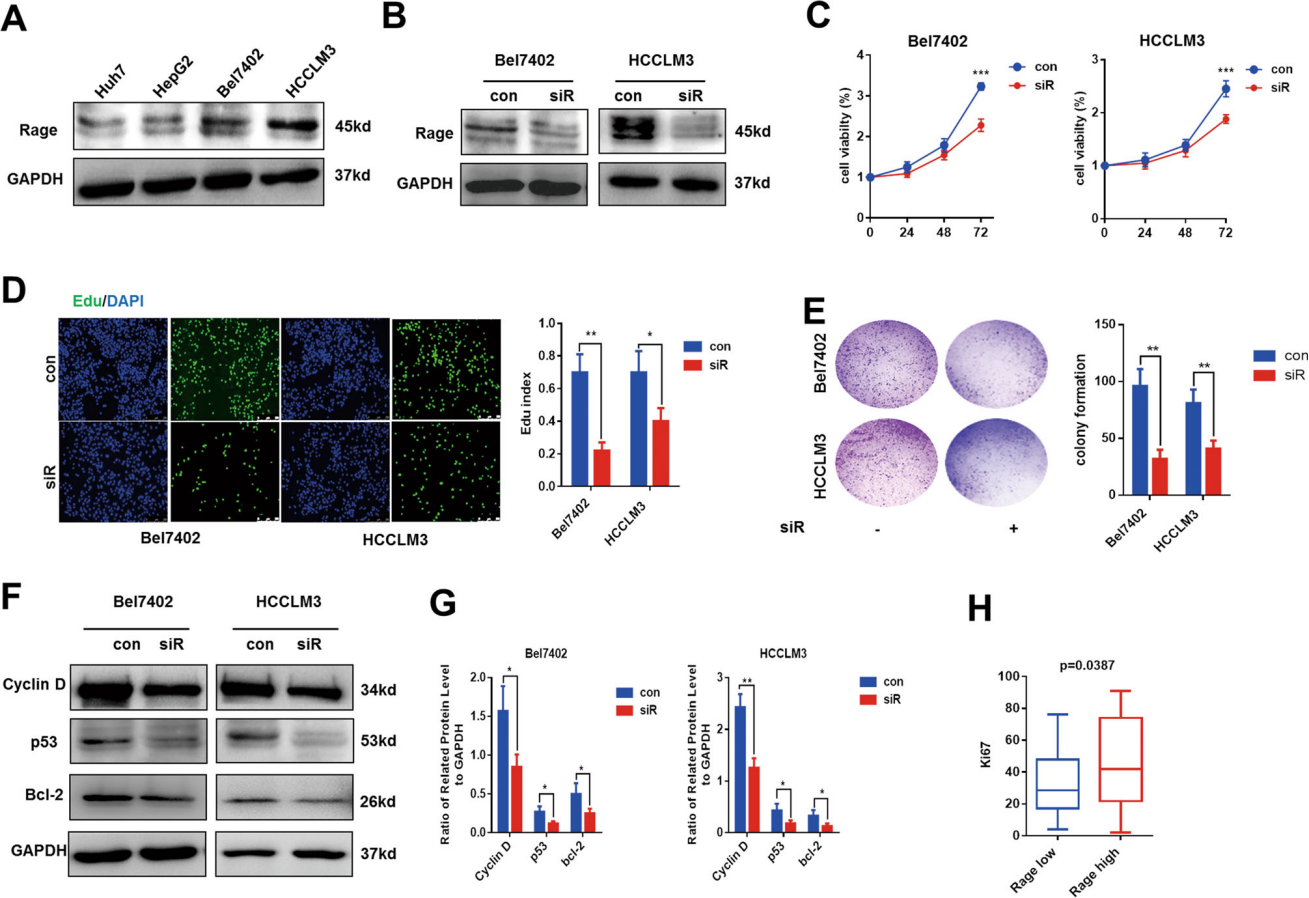
Rage promotes proliferation of HCC. **a** Immunoblot analysis of different expression of Rage in four HCC cell lines. **b** Immunoblot analysis confirmed the downregulation of Rage in cells treated with Rage siRNA. **c** Rage ablation inhibited proliferation of HCC cells. **d** Edu assays were performed and analyzed. **e** Colony formations were performed and analyzed. **f**,** g** Rage ablation contributed to reduction of cyclin D, p53, and Bcl-2. Protein levels of cyclin D, p53, and Bcl-2 were normalized to GAPDH and analyzed. **h** Rage expression was positively associated with Ki67 in HCC samples. Data are mean ± SEM, *p < 0.05, **p < 0.01, ***p < 0.001 by unpaired Student’s *t-*test

We evaluated Rage expression in four HCC cell lines, finding different protein levels of Rage in various cell lines (Fig. 2a, Fig. S2). To explore the role of Rage in HCC development, Rage siRNA was well designed and Rage-deficient cells (Bel7402 siR and HCCLM3 siR) were established (Fig. 2b). We found that, compared to normal cells, the proliferation of Bel7402 siR and HCCLM3 siR was significantly decreased (Fig. 2c). Moreover, edu assays and colony formation experiments were performed and results were similar, which enhanced the role of Rage in proliferation of HCC cells. Proliferation-related proteins, such as p53, Cyclin D, and Bcl-2 were all downregulated in Rage ablation cells. To further investigate the role of Rage in proliferation, immunohistochemistry staining Ki67 were conducted in HCC tissues and analyzed. We demonstrated that there was a positive correlation between Rage and Ki67. These finding indicated that high expression of Rage promoted the proliferation of HCC cells.

### Autophagy is involved in Rage-mediated proliferation

**Fig. 3:**
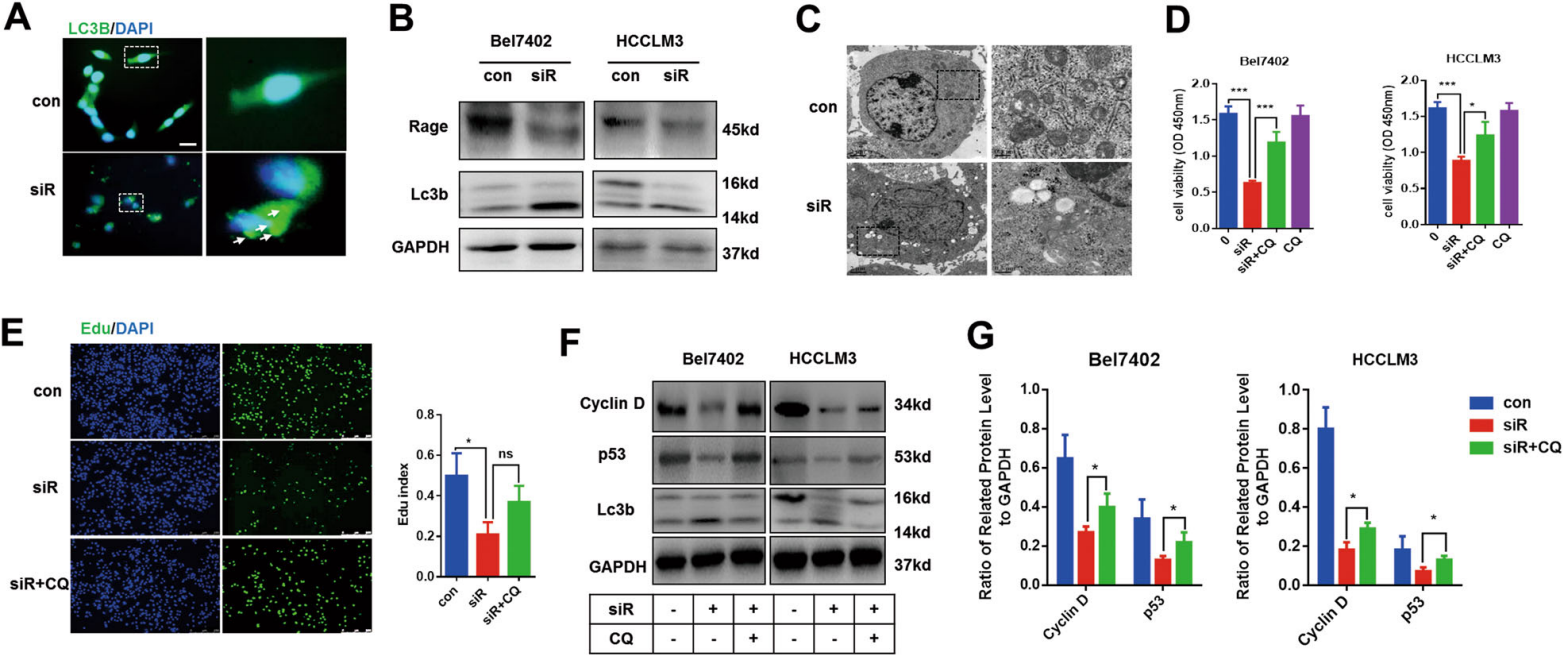
Knockdown of Rage results in autophagy induction in HCC cells. **a** Immunofluorescence staining lc3b were performed. **b** Immunoblot analysis of lc3b and Rage. **c** Autophagesomes were shown by transmission electron microscopy. **d**, **e** Autophagy induction caused by Rage deficiency was responsible for proliferation inhibition. CCK8 and edu assays were performed and analyzed. CQ (50 μM) was used to inhibit autophagy induction. **f**, **g**Immunoblot analysis shows that autophagy induction caused by Rage deficiency accounts for decrease of cyclin D and p53. Protein levels of Cyclin D and p53 were normalized to GAPDH and analyzed. CQ (50 μM) was used to inhibit autophagy induction. Data are mean ± SEM, *p < 0.05, **p < 0.01, ***p < 0.001 by unpaired Student’s *t*-test

Recent studies reported that Rage promoted tumor cells survival via sustaining autophagy^15^. Autophagy has been demonstrated to be involved in tumor progression^7^. Stress-induced autophagy could promote cell survival, but excessive autophagy would contribute to apoptosis^8,17^. To visualize the change of autophagy, immunofluorescence staining lc3b were performed. Accumulating of lc3 puncta was observed in Rage ablation cells, which suggested that Rage deficiency could increase the level of autophagy (Fig. 3a). In addition, the ratio of lc3b/lc3a in Rage knockdown cells was higher than normal cells, which confirmed the findings that autophagy was induced in Rage-deficient cells (Fig. 3b). Using transmission electron microscope (TEM), we observed more autophagesomes characterized by double-membrane vehicles engulfing cellular organelles in Rage ablation cells (Fig. 3c). To determine the relationship between autophagy and impaired proliferation both caused by Rage ablation, Bel7402 siR and HCCLM3 siR were treated with chloroquine (CQ), an autophagy inhibitor, for 24 h. With the treatment of CQ, we found that the impairment of proliferation in Rage ablation cells was partly rescued, which indicated that autophagy induction was responsible for proliferation repression (Fig. 2d). Similar results were got from edu assays (Fig. 2e). Moreover, we found repression autophagy in Rage ablation cells via CQ re-expressed the expressions of p53 and Cyclin D (Fig. 3f, g). These data suggested that autophagy induction caused by Rage deficiency accounted for proliferation inhibition.

### AMPK/mTOR is essential for autophagy induction in Rage-deficient cells

**Fig. 4:**
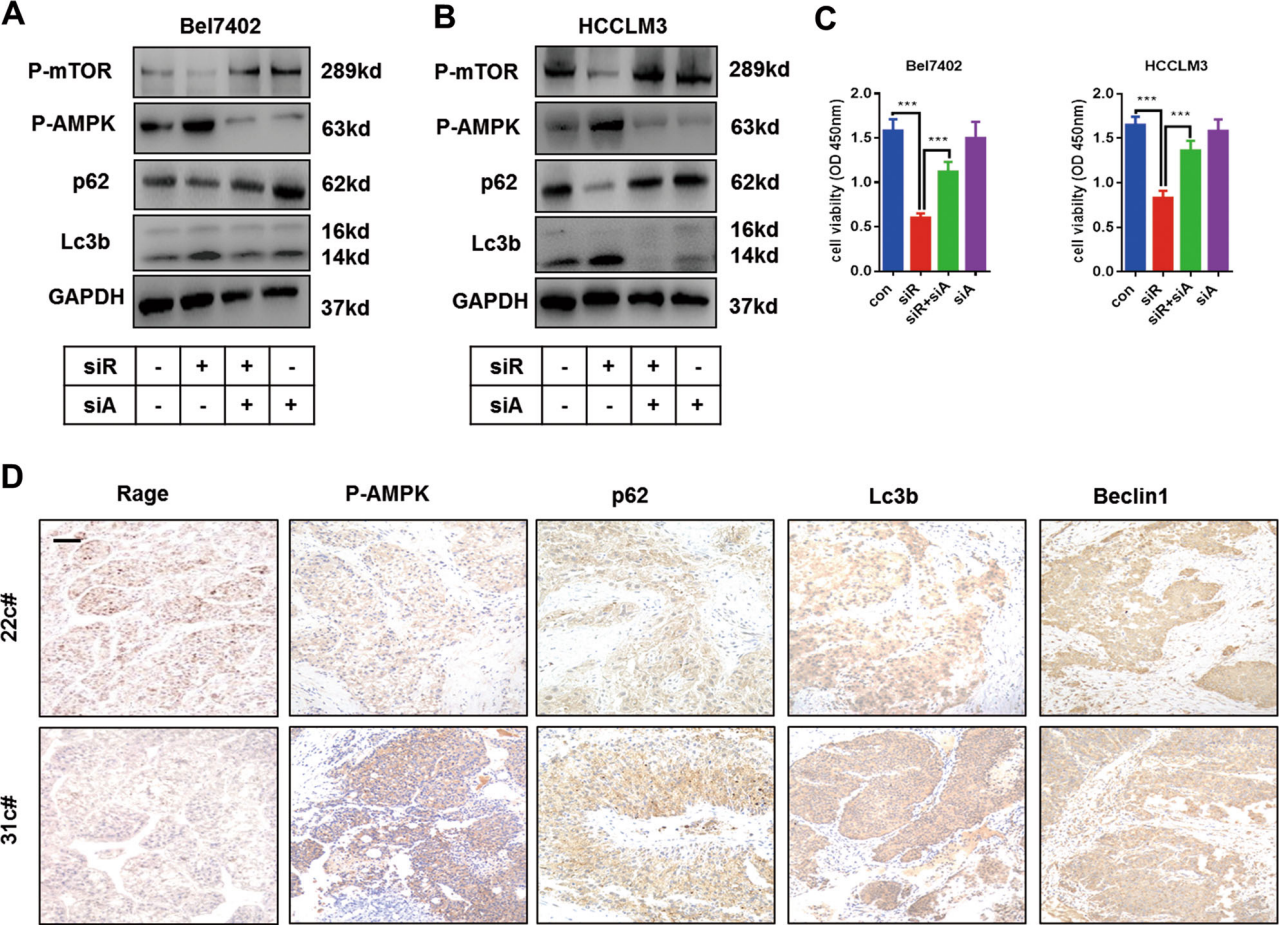
Activation of AMPK/mTOR signaling is essential for autophagy induction and proliferation repression in Rage ablation cells. **a**, **b** Autophagy induction caused by Rage ablation was dependent on AMPK/mTOR signaling pathway. **c** Proliferation repression caused by Rage ablation was dependent on AMPK/mTOR signaling pathway. CCK8 assays were conducted and analyzed. **d** IHC of Rage, p-AMPK, beclin-1, lc3b, and p62 in HCC samples. Representative images show different intensities of staining of Rage, p-AMPK, beclin-1, lc3b, and p62. Scale bars, 100 μm. Data are mean ± SEM from three independent experiments, *p < 0.05, **p < 0.01, ***p < 0.001 by unpaired Student’s *t-*test

Many signaling pathways have been reported to be involved in autophagy induction, including PI3K/AKT/mTOR and AMPK/mTOR^18–21^. mTOR has been demonstrated to inhibit the ULK1complex, which was essential for autophagy induction at an early stage^22^. To examine the relationship between Rage and mTOR, immunohistochemistry staining p-mTOR and Rage were performed. We found that patients with high expression of Rage are characterized with high expression of p-mTOR (Fig. S2A). Moreover, in vitro experiments, we observed that PI3K/AKT and NF-κB signaling pathway were repressed, but AMPK signaling was activated (Fig. S2B). Both inactivation of PI3K/AKT and activation of AMPK are responsible for p-mTOR inhibition. To test the role of AMPK in Rage ablation cells, Bel7402 siR and HCCLM3 siR cells were treated with AMPK siRNA to silence AMPK expression. With the silence of AMPK, the decrease of p-mTOR and p-AKT was partly rescued, indicating that AMPK signaling played a more important role in Rage-deficient cells (Fig. S3C). Moreover, we found that silencing AMPK could repress autophagy induction caused by Rage ablation (Fig. 4a, b). Following the decrease level of autophagy, cell proliferation abilities of Rage-deficient cells were partly recovered (Fig. 4c). To further determine the relationship between Rage and AMPK/mTOR signaling, we performed immunohistochemistry staining relevant proteins and demonstrated the inverse correlation of Rage and AMPK/mTOR activation (Fig. 4d). These findings suggested that Rage deficiency-mediated autophagy induction was dependent on AMPK/mTOR signaling pathway.

### Rage induces sorafenib resistance in HCC cells by modulating AMPK/mTOR signaling pathway

As a multi-kinase inhibitor, sorafenib has been proven to an effectively systematic treatment for advanced HCC. However, sorafenib treatment has demonstrated to be with a low response rate and acquired resistance of sorafenib obviously limited survival benefits. Recent studies showed that alternative activation of mTOR signaling was responsible for sorafenib resistance^10,15^. As reportedly, we found AMPK activation and mTOR inhibition in three HCC cell lines treated with sorafenib (Fig. S4A). To determine the role of AMPK/mTOR in sorafenib response, AMPK siRNA was used to repress AMPK activation in sorafenib-treated cells. With the inactivation of AMPK, we demonstrated that sorafenib-induced apoptosis was suppressed, which suggested that activation of AMPK was essential for sorafenib response (Fig. S4B–C). Moreover, Rapamycin or Metformin treatment both targeting inhibition of mTOR could significantly enhance sorafenib response (Fig. S4D).

**Fig. 5:**
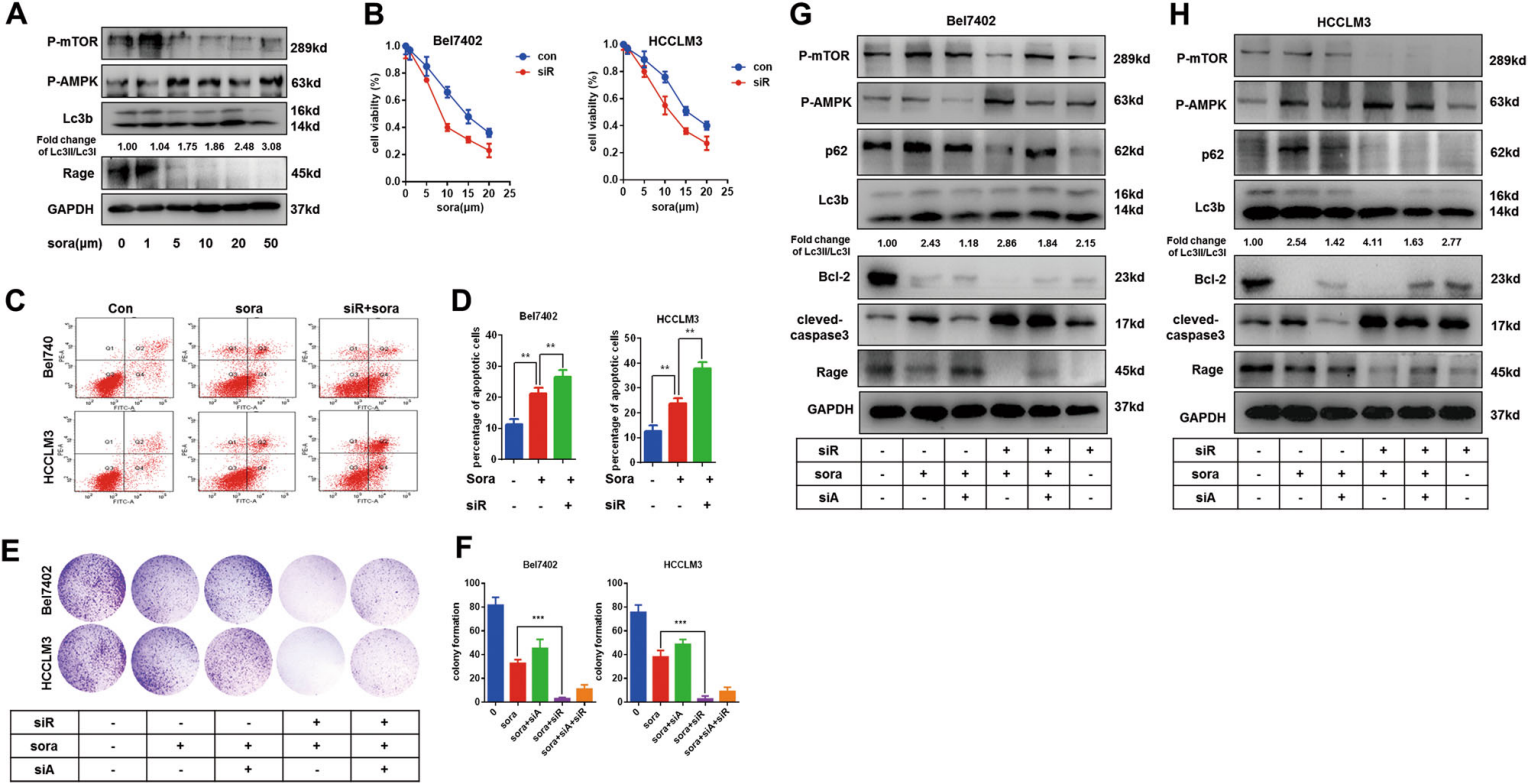
Targeting Rage enhances sorafenib response via activating AMPK/mTOR signaling pathway. **a** Immunoblot analysis reveals that sorafenib inhibited Rage expression and activated AMPK/mTOR pathway. HCCLM3 cells were treated with different concentration of soarfenib for 24 h. Autophagy activity was measured by analyzing the fold change of Lc3II/Lc3I. **b** Rage ablation enhanced sorafenib response. CCK8 assays were performed and analyzed. **c**,** d** Apoptosis assays were conducted and analyzed. Bel7402 and HCCLM3 cells were cultured with sorafenib (10 μM) for 24 h. **e**, **f** Colony formation experiments were conducted and analyzed. Bel7402 and HCCLM3 cells were cultured with sorafenib (10 μM) for 24 h. **g** Immunoblot analysis indicates that targeting Rage strengthened sorafenib response, which was dependent on AMPK/mTOR pathway. Bel7402 and HCCLM3 cells were cultured with sorafenib (10 μM) for 24 h. Autophagy activity was measured by analyzing the fold change of Lc3II/Lc3I. Data are mean ± SEM from three independent experiments, *p < 0.05, **p < 0.01, ***p < 0.001 by unpaired Student’s *t-*test

Former studies confirmed that Rage promoted HCC proliferation via regulating AMPK/mTOR pathway. To explore whether Rage was involved in sorafenib response, HCC cells were cultured with varied concentration of sorafenib. Interestingly, we found that sorafenib treatment contributed to Rage decrease and activation of AMPK/mTOR in a dose-dependent manner (Fig. 5a). Moreover, compared to normal cells, IC50 of sorafenib treatment in Rage-deficient cells was markedly decreased (Fig. 5b). Rage inhibition not only strengthened sorafenib causing apoptosis but also proliferation repression (Fig. 5c–f). Moreover, AMPK silencing partly recovered impaired proliferation caused by sorafenib treatment in Rage ablation cells (Fig. 5e, f). To unveil the underlying molecular mechanism of Rage in sorafenib response, western blot was performed. We demonstrated that Rage ablation elevated autophagy levels in HCC cells treated by sorafenib through further activating AMPK/mTOR signaling and silencing AMPK could inhibited autophagy process and then partly rescued proliferation repression as well as apoptosis induction caused by sorafenib (Fig. 5g, h). These findings suggested Rage participated in sorafenib response by regulating autophagy level, which was dependent on AMPK/mTOR pathway.

### Rage expression is enhanced by its ligands, such as HMGB1 and s100a4

**Fig. 6:**
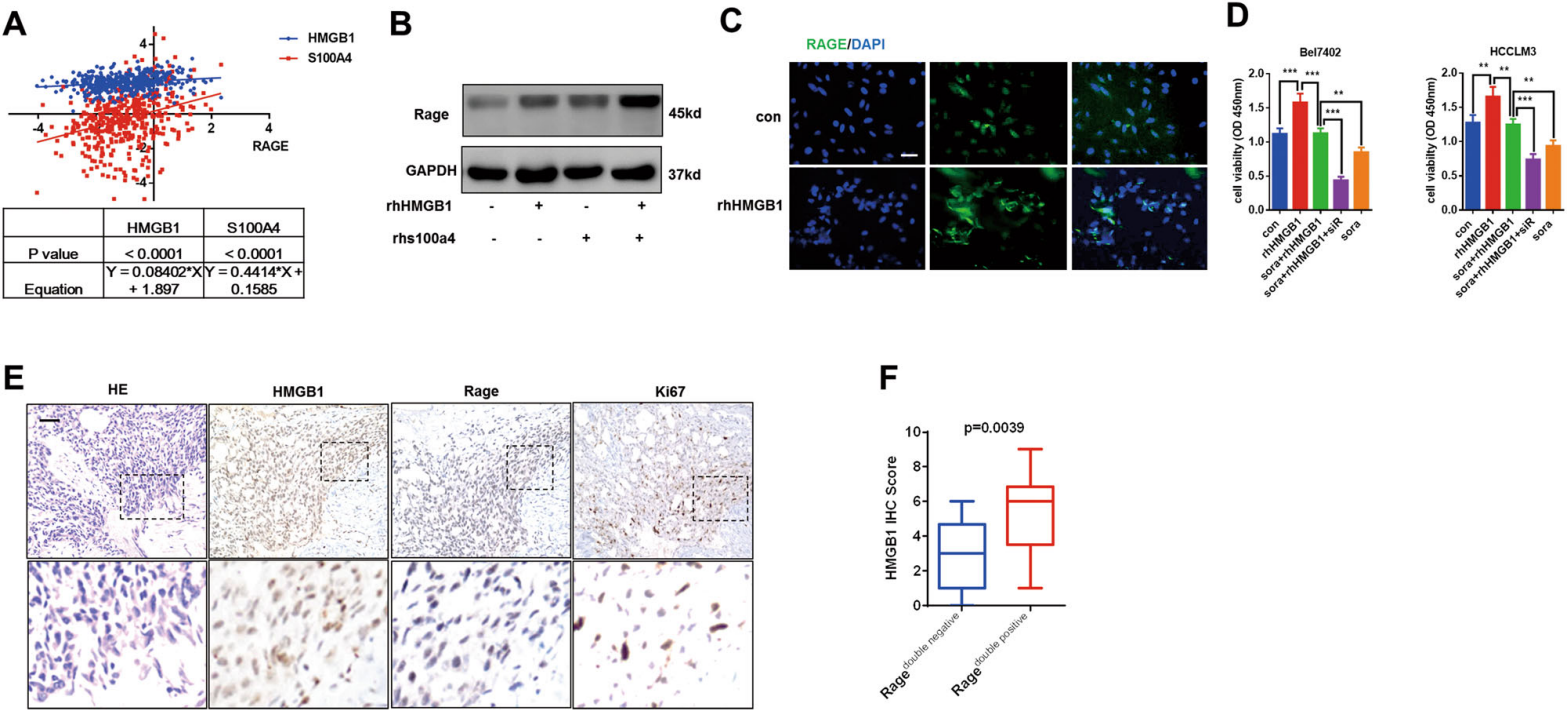
The interactions of Rage ligands and Rage promote Rage expression and repress sorafenib response. **a** Rage expression was positively associated with Rage ligands, sush as HMGB1 and s100a4. Data were extracted from TCGA database (https://xenabrowser.net/heatmap/). **b** HMGB1 and s100a4 promoted Rage expression and activated AMPK/mTOR pathway. **c** Rage expression was analyzed through immunofluorescence. Bel7402 cells were cultured with HMGB1 (1 μg/mL) for 24 h. **d** HMGB1 repressed sorafenib response, which was dependent on Rage. CCK8 assays were conducted and analyzed. **e** IHC of Rage, HMGB1, and ki67 in HCC samples. Representative images show different intensities of staining of Rage, HMGB1, and ki67. Scale bars, 100 μm. **f** HMGB1 expression was positively associated with Rage expression. IHC of Rage, HMGB1, and ki67 in HCC samples were analyzed. Data are mean ± SEM from three independent experiments, *p < 0.05, **p < 0.01, ***p < 0.001 by unpaired Student’s *t*-test

The interactions between Rage and its ligands, such as HMGB1 and s100a4, play a critical role in tumor progression through activating MAPK, NF-κB, and producing inflammatory cytokines^12^. Accumulating data demonstrated that HMGB1 and s100a4 were both involved in inflammatory diseases including tumors and inflammatory factors could contributed to the production of HMGB1 and s100a4^23–26^. Through analyzing expression of HMGB1, s100a4, and Rage in TCGA data, we found there was a positive correlation between Rage and its ligands (Fig. 6a). To determine the role of Rage ligands in Rage expression, HCC cells were treated with rhHMGB1 and rhs100a4. Data suggested that the interactions of Rage and its ligands resulted in Rage increase (Fig. 6b, c). Moreover, we demonstrated that Rage ligand HMGB1 could promote proliferation and repress sorafenib response in a Rage-dependent way (Fig. 6d). To further exploring the relationship between Rage and HMGB1, immunohistochemistry staining HMGB1, Rage, and Ki67 were performed and quantified. We observed HMGB1 was positively associated with Rage and proliferation (Fig. 6e, f). These observations indicated the important role of Rage ligand HMGB1 in Rage expression.

### Rage induces sorafenib resistance in vivo

**Fig. 7:**
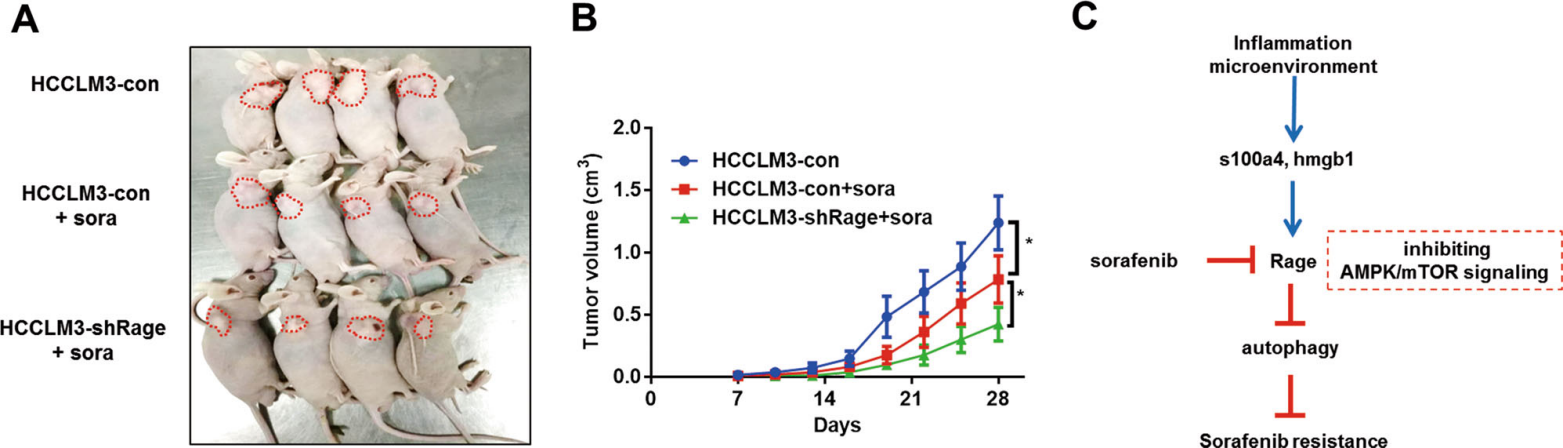
Targeting Rage enhances sorafenib response in vivo. **a** 5 × 10^6^ HCCLM3-con, HCCLM3-shRage cells were subcutaneously injected into nude mice and those mice were treated with or without sorafenib (50 mg/kg/d). Four weeks later, tumors derived from indicated cells were removed and shown, *n* = 4. **b** The tumor growth curves for three groups as described in **a** were measured. **c** Our study suggested that Rage was elevated in tumors and regulates proliferation via modulating AMPK/mTOR pathway. Autophagy induction caused by Rage ablation was dependent on AMPK/mTOR signaling. Moreover, soarfenib response was enhanced by downregulation of Rage whose repression activated AMPK/mTOR pathway. Lastly, we found Rage ligands, especially HMGB1, promoted Rage expression and decreased sorafenib response through inactivating AMPK/mTOR pathway

To further confirm the crucial role of Rage in sorafenib resistance, HCCLM3-shRage cells were well established and tumor xenografts and orthotopic models in nude mice were performed. Compared to mice bearing HCCLM3 cells, tumor growth inhibition caused by sorafenib treatment in those bearing HCCLM3-shRage cells was more significant (Fig. 7a, b, Fig. S5). Taken together, our study provided evidence that Rage ablation inhibited HCC proliferation via regulating AMPK/mTOR pathway, which was responsible for autophagy induction. Moreover, targeting Rage could enhance sorafenib response by increasing autophagy level. Lastly, Rage ligands were important for Rage expression and alleviate sorafenib response in a Rage-dependent manner.

## Discussion

Accumulating data demonstrate that Rage is overexpressed in various types of tumors and has a vital role tumor progression^12,14^. Recent studies reported that Rage promotes proliferation via activating NF-κB/cyclin D pathway^13^. Moreover, targeting Rage could enhance chemotherapeutic effects and promote tumor cell survival by reducing autophagy^16^. Our study suggested that Rage was expressed in both tumor and stromal cells and confirmed that Rage expression was highly elevated in tumors. We observed that targeting Rage indeed contributed to autophagy induction in HCC cell lines. Mounting evidence has been reported to show the paradox role of autophagy in pro-survival and pro-apoptosis^27^. Our study demonstrated that autophagy induction caused by Rage deficiency was responsible for proliferation impairment in HCC cells. Recent studies indicate that Rage ablation results in increase of phosphorylation of mTOR in pancreatic tumor cells, which is responsible for Rage-sustained autophagy^15,16,28^. In our study, we confirmed that activation of AMPK/mTOR pathway in Rage ablation cells accounted for autophagy increase and proliferation impairment, which might explain the different relationship between autophagy and Rage in varied tumor cells. More efforts are needed to be performed to explore the underlying mechanisms of Rage and AMPK/mTOR pathway.

The detailed molecular basis for acquired sorafenib resistance remains complicated. Many cellular signaling pathways have been proven to be involved in sorafenib-resistant cells. Emerging evidence demonstrated that alternative activation of mTOR accounts for sorafenib resistance in varied cells, including AML and HCC^9,10^. Inhibition of mTOR is a crucial step for autophagy process, which promotes the formation of ULK complex^18,29^. Moreover, the role of autophagy in sorafenib therapy is still controversial^30^. A lot of studies indicate that sorafenib-induced autophagy was responsible for tumor survival, which inhibited the cytotoxic effects of sorafenib treatment. Combining autophagy inhibitors and sorafenib could enhance sorafenib response^27,31–33^. However, contrary results were also reported. Sorafenib was demonstrated to promote autophagic death of HCC^34^. Additionally, activation of ARDB2 was confirmed to promote sorafenib resistance by inhibiting autophagy, which suggested that autophagy inhibition might be a potential therapy for enhancing sorafenib response^9^. Our study showed Rage ablation elevated autophagy levels through activating AMPK/mTOR signaling pathway. We firstly reported that Rage decrease and AMPK/mTOR activation in sorafenib-treated HCC cells. We found inhibition of AMPK impaired sorafenib response and combination of Rapamycin or Metformin, both targeting repression of mTOR, strengthened it^35^. Moreover, Rage ablation increased the efficiency of sorafenib-induced apoptosis in a AMPK manner, which suggested that Rage was involved in sorafenib response through regulating AMPK activity.

The interactions of Rage and its ligands have been reported to promote tumor progression by activating MAPK, NF-κB, PI3K/AKT, and cdc42 pathways^14^. Our study demonstrated that Rage ligands including HMGB1 and s100a4 significantly increased Rage expression. Additionally, HMGB1 treatment inhibited sorafenib response in a Rage-dependent way. Accumulating data demonstrated that inflammatory microenvironment of HCC played an important role in sorafenib response^36^. Recent studies indicated that patients with less inflammation benefited more from sorafenib therapy^36^. Rage ligands and Rage were tightly associated with inflammation, which indicated that inflammatory microenvironment of HCC might repress sorafenib response and resulted in sorafenib resistance through regulating Rage expression and stimulating the interactions between Rage and its ligands.

In conclusion, we demonstrated the critical role of Rage in HCC proliferation. Autophagy induction caused by Rage ablation via activating AMPK/mTOR pathway was responsible for impaired proliferation. Moreover, Rage was involved in sorafenib response through modulating AMPK activity and Rage ablation significantly increased sorafenib-induced apoptosis. Lastly, we observed that HMGB1 treatment, as a Rage ligand, not only enhanced Rage expression but contributed to repression of sorafenib repsonse. Consequently, our study indicated targeting Rage would be a potential therapy for HCC and sorafenib resistance.

## Material and methods

### Patients and specimens

Samples were achieved from 68 HCC patients who had undergone curative resection between 2014 and 2017 and were pathologically confirmed HCC at Medical School of Nanjing University Affiliated Drum Tower Hospital. Informed consent was obtained from each recruited patient, and the protocol was approved by the Institutional Research Ethics Committee. The clinical signatures of all patients are summarized in Supplementary Table 1.

### Animals and chemical reagents

Male BALB/c nu/nu mice (6–8 weeks old, Shanghai Institute of Material Medicine, Chinese Academy of Science) were housed in specific pathogen-free conditions. All animals received humane care according to the criteria outlined in the “Guide for the Care and Use of Laboratory Animals” prepared by the National Academy of Sciences and published by the National Institutes of Health (NIH publication 86–23 revised 1985). Sorafenib (No. S7397), Rapamycin (No. S1039), and Metformin (No. S1950) were purchased from Selleck Chemicals (Houston, TX, USA). The PI (propidiumiodide)/Annexin V-FITC apoptosis detection kit was from BD Biosciences (San Jose, CA, USA). The Cell Counting Kit-8 (CCK-8) kit was purchased from Dojindo Laboratories (Kumamoto, Japan). Edu assays (KGA331-100) were purchased from KeyGENE BioTECH (Nanjing, China).

### Antibodies

The antibody catalog numbers were provided in Supplementary Table 2.

### Cell culture

The human HCC cell line HepG2, HCCLM3, Huh7, Bel7402 was achieved from the Cell Bank of the Chinese Academy of Sciences (Shanghai, China). Four HCC cells were al cultured in 4.5 g/L glucose DMEM containing 10% FBS (Gbico, USA) and 10% FBS, penicillin (100 U/mL), and streptomycin (100 μg/mL). LO2 cells were cultured in RPMI1640 medium containing 10% FBS, penicillin (100 U/mL), and streptomycin (100 μg/mL). All cells were incubated at 37 °C in humidified air with 5% CO_2_.

### Immunoblot analysis

Total protein was extracted by lysing cells in RIPA buffer containing protease inhibitor cocktail. Protein samples boiled with 1× loading buffer were separated by sodium dodecyl sulfate polyacrylamide gel electrophoresis and transferred onto polyvinylidene fluoride membranes. After blocking with 5% BSA in TBS-T, membranes were incubated with the primary antibody at 4 °C overnight. Goat-anti-rabbit or mouse IgG conjugated to horseradish peroxidase was used as the secondary antibody. Protein was imaged by Tanon System.

### Cell transfection

For stably knockdown of Rage with lenti-virus shRNA, 2 × 10^5^ cells were planted onto 6-well plates. After 24 h, the liquid containing shRNA was added to cultural medium according protocol. To select stable transfectants, cells were cultured in complete DMEM with 10 μg/mL puromycin (Sigma-Aldrich, USA) for some weeks. AMPK siRNA, Rage siRNA, and control siRNA (RiboBio, China) were transfected into cells using lipofectin 2000 according to the manufacturer’s instructions. At the end of the siRNA treatment (48–72 h), the cells were collected for western blot and q-PCR.

### Immunofluorescence

Immunofluorescence analysis was performed according to protocols. Cells were implanted in 24-well dishes and fixed by 4% paraformaldehyde 24 h later. Fixed cells were stained with autophagy-related proteins (Cell Signaling Technology, USA), Rage (Cell Signaling Technology), followed by FITC-conjugated anti-mouse IgG and Cy3-conjugated anti-rabbit IgG (Abcam). Representative images were detected by fluorescent microscopy (Leica, German) and data were processed via ImagePro Plus.

### Immunohistochemistry staining

Immunohistochemistry of HCC samples were performed as previously described. Briefly, after incubation with HMGB1 (Abcam), Rage (Abcam), autophagy-related markers (Cell Signaling Technology, USA), p-AMPK (Cell Signaling Technology, USA), mTOR (Cell Signaling Technology, USA), the sections were stained in an Envision System (Dako Cytomation, USA). IHC results were scored according to 0, <25%; 1, <50%; 2, <75%; 3, >75% by two experienced pathologists. Data are shown as mean ± SEM.

### Cell proliferation

Cell proliferation was tested by Cell Counting Kit-8 and edu assays. According to manufacturer’s instructions, cells were seeded in 96-well plates at 5 × 103 per well. Cells were cultured with different treatments for 24, 48, and 72 h. 10 μL CCK-8 solutions was added into each well, and after incubation for 2–4 h at 37 °C, the absorbance at 450 nm for each well was measured to estimate the number of viable cells. For edu assays, all performances were according to manufacturer’s instructions.

### Transmission electron microscopy

Cells seeded onto 6-well plate were fixed with fixative buffer containing 2% paraformaldehyde and 2.5% glutaraldehyde in 0.1 M PBS. After embedded, samples were cut into 0.12-μm sections and stained with 0.2% lead citrate and 1% uranyl acetate. The images were detected by a JEOL TEM-2000 EX II (JEOL, Tokyo, Japan).

### Statistical analysis

Fisher’s exact tests and *χ*^2^ tests were used to determine clinicopathological correlations. The association between Rage, autophagy markers, and p-mTOR in HCC tissues was evaluated by Spearman’s correlation. The Student’s *t*-test was used for comparison between variables. GraphPad Prism 6 was used for all statistical analyses. *P* < 0.05 was considered statistically significant.

## Electronic supplementary material

Fig.S1

Fig.S2

Fig.S3

Fig.S4

Fig.S5

supplement

Supplementary Figure Legends
