# Supplementary material for: Rage induces hepatocellular carcinoma proliferation and sorafenib resistance by modulating autophagy
Source: Cell Death Dis. 2018 Feb 14;9(2):225. doi: 10.1038/s41419-018-0329-z (PMC5833717; doi:10.1038/s41419-018-0329-z)
Supplement: Supplementary file 6 — supplement [file 41419_2018_329_MOESM6_ESM.docx]

**Supplementary Tables**

| **Supplementary Table 1: The associations of Rage expression with clinicopathological characteristics in HCC patients** | | | |  |
| --- | --- | --- | --- | --- |
| Feature | Low expression of Rage(n=40) | Hgh expression of Rage(n=28) | P-value |  |
| Gender |  |  |  |  |
| Male | 32 | 19 | 0.25^a^ |  |
| Female | 8 | 9 |  |  |
| Age(years) |  |  |  |  |
| >=50 | 30 | 17 | 0.21 |  |
| <50 | 10 | 11 |  |  |
| HBsAg |  |  |  |  |
| Negative | 15 | 6 | 0.19^a^ |  |
| Positive | 25 | 22 |  |  |
| AFP(ng/ml) |  |  |  |  |
| >=20 | 24 | 20 | 0.33 |  |
| <20 | 16 | 8 |  |  |
| Cirhosis |  |  |  |  |
| No | 24 | 11 | 0.05 |  |
| Yes | 16 | 19 |  |  |
| Tumor size(cm) | |  |  |  |
| >=5 | 22 | 21 | 0.09 |  |
| <5 | 18 | 7 |  |  |
| Tumor number |  |  |  |  |
| Single | 26 | 16 | 0.51 |  |
| Multiple | 14 | 12 |  |  |
| PVTT |  |  |  |  |
| No | 27 | 24 | 0.08^a^ |  |
| Yes | 13 | 4 |  |  |
| TNM stage |  |  |  |  |
| I+II | 27 | 12 | 0.04 |  |
| III+IV | 13 | 16 |  |  |
| Recurrence |  |  |  |  |
| No | 35 | 22 | 0.32 |  |
| Yes | 5 | 6 |  |  |
| Abbreviations: AFP, α -fetoprotein; HBsAg, hepatitis B surface antigen; HCC, hepatocellular carcinoma; Rage, the receptors of advanced glycation ends products; ^a^Fisher’s exact tests and Pearson's χ ^2^ tests for all the other analysis. | | | |  |

**Supplementary Table 2:** The antibody catalog numbers
