# Supplementary material for: Rage induces hepatocellular carcinoma proliferation and sorafenib resistance by modulating autophagy
Source: Cell Death Dis. 2018 Feb 14;9(2):225. doi: 10.1038/s41419-018-0329-z (PMC5833717; doi:10.1038/s41419-018-0329-z)
Supplement: Supplementary file 7 — Supplementary Figure Legends [file 41419_2018_329_MOESM7_ESM.docx]

**Supplementary Figure Legends**

Fig.S1 Rage expression was enhanced in tumors

(A-B) IHC of Rage in HCC samples. Different intensities of staining of Rage in tumor cells were represented and analyzed. Scale bars, 100μm.

Fig.S2 Different expression of Rage in four HCC lines

(A) Rage expression in Huh7, HepG2, Bel7402 and HCCLM3 cells were measured by immunoblot analysis. An obvious band was shown around 45kd.

Fig.S3 Correlation of Rage and p-mTOR in HCC samples

(A) IHC of Rage and p-mTOR in HCC samples. Representative images show different intensities of staining of Rage and p-mTOR in tumor cells. Scale bars, 100μm. (B) Immunoblot analysis shows that changed signaling pathway in Rage deficient cells.

Fig.S4 AMPK/mTOR pathway is activated by sorafenib and accounts for sorafenib response

(A) Immunoblot analysis shows sorafenib contributed to activation of AMPK/mTOR pathway and Rage decrease. Bel7402, HepG2 and HCCLM3 cells were cultured with sorafenib (10μM) for 24h. (B-C) Apoptosis assays were conducted and analyzed. (D) Combination of sorafenib and Rapamycin (Rapa, 1μM) or Metformin (M, 10mM) respectively resulted in elevation of sorafenib response. CCK8 assays were performed and analyzed. Data are means ± SEM, * means p<0.05, ** means p<0.01, *** means p<0.001 by unpaired student T test.

Fig.S5 Rage deficiency enhances sorafenib response in vivo

(A) HCC Orthotopic models were perfoermed. HCCLM3-con, HCCLM3-shRage cells were subcutaneously injected into nude mice. After 15 days, the tumor mass was removed and minced into small pieces (2×2×2 mm3), and transplanted into the livers of normal nude mice. Those nude mice were divided into three groups including HCCLM3-con, HCCLM3-con+sora and HCCLM3-shRage+sora. HCCLM3-con+sora and HCCLM3-shRage groups were treated with sorafenib (50mg/kg/d). Four weeks after implantation, the livers bearing tumors were obtained and shown. n = 4.
